# Undergraduate students’ mental health in the first year of the COVID-19 pandemic: a scoping review

**DOI:** 10.1590/0034-7167-2024-0032

**Published:** 2025-03-10

**Authors:** Fabio Araujo Dias, Eliane Ramos Pereira, Rose Mary Costa Rosa Andrade Silva, Angelica Yolanda Bueno Bejarano Vale de Medeiros, Janaina Mengal Gomes Fabri

**Affiliations:** I Universidade Federal Fluminense. Niterói, Rio de Janeiro, Brazil

**Keywords:** Mental Health, Pandemic, COVID-19, Students, Health, Salud Mental, Pandemia, COVID-19, Estudiantes, Salud

## Abstract

**Objectives:**

to map evidence on undergraduate students’ mental health globally during the COVID-19 pandemic.

**Methods:**

a scoping review, in which PRISMA-ScR was used.

**Results:**

twenty-six articles were included, from which data were collected on the characteristics of articles, participants involved, and results. The results were categorized into: Undergraduate students’ mental health; Stressors and factors associated with mental health problems; Impact of spirituality and meaning in life on students’ mental health; Relationship between physical activity and mental health; Mental health and digital education.

**Final Considerations:**

the pandemic has intensified the mental health challenges faced by undergraduate students, highlighting the need for strategic interventions. It is suggested that educational institutions implement psychological support programs, encourage healthy practices, spirituality, and the search for meaning. It is clear that such measures can mitigate the negative effects of the pandemic and strengthen students’ resilience.

## RESUMEN

**Objetivos:**

mapear la evidencia sobre la salud mental de estudiantes de pregrado en un contexto global durante la pandemia de COVID-19.

**Métodos:**

revisión del alcance, en la que se utilizo PRISMA-ScR.

**Resultados:**

se incluyeron 26 artículos, de los cuales se recogieron datos sobre las características de los artículos, participantes involucrados y resultados. Los resultados fueron categorizados en: Estado de salud mental de estudiantes universitarios; Factores estresantes y asociados con problemas de salud mental; Impacto de la espiritualidad y el significado de la vida en la salud mental de los estudiantes; Relación entre actividad física y salud mental; Salud mental y enseñanza digital.

**Consideraciones Finales:**

la pandemia ha intensificado los desafíos de salud mental que enfrentan los estudiantes universitarios, destacando la necesidad de intervenciones estratégicas. Se sugiere que las instituciones educativas implementen programas de apoyo psicológico, fomenten prácticas saludables, la espiritualidad y la búsqueda de sentido. Está claro que este tipo de medidas pueden mitigar los efectos negativos de la pandemia y fortalecer la resiliencia de los estudiantes.

## INTRODUCTION

In December 2019, in Wuhan, Hubei province, China, there were the first reports of the emergence of a new pneumonia caused by the novel coronavirus (SARS-CoV-2), giving rise to the d isease that was named COVID-19^(1)^. Authors^(2)^ pointed out that the prevalence of epidemics accentuates and creates new stressors. In addition to fear, worry and sadness in the face of a health crisis of this magnitude, the population faces restrictions on physical movement and social activities as well as radical and sudden changes in lifestyle, impacting individuals’ mental health, their spirituality and meaning of life^(3)^.

The pandemic has affected specific populations in different ways. Thus, we mentioned here undergraduate students, who are also known as a vulnerable population during this pandemic period and about whom there is still a lack of studies related to the psychological effects of the disease on their mental health^(4)^. Research indicates that undergraduate students often already face psychological issues, such as anxiety, panic and depression, due to the complications of higher education courses. With the pandemic, new complications arise. These include closed educational institutions, lost semesters, postponed theses, replacement of in-person teaching with remote teaching and more^(3)^.

Thus, the following question was formulated: what is the scientific evidence regarding undergraduate students’ mental health in the first year of the pandemic and the intervention proposals adopted during this period?

It is expected to contribute to current knowledge on undergraduate students’ mental health, enriching the field of public health with data on the repercussions of COVID-19 on undergraduate students’ lives and mental health in the first year of the pandemic period, and on the interventions applied in that context.

In this regard, this study, by identifying students’ needs, bringing an understanding of the impacts of the pandemic, identifying risk factors and proposed interventions and making a comparison among countries, proves to be relevant in contributing to guiding efforts to mitigate the impacts and improve these young people’s quality of life, developing more effective and personalized prevention and intervention strategies.

## OBJECTIVES

To map evidence on undergraduate students’ mental health globally during the COVID-19 pandemic.

## METHODS

### Ethical aspects

The studies included in this scoping review demonstrate concern with the ethical aspects involved in research with human participants. Thus, they presented an approach to ensure participant integrity protection, following the ethical guidelines established by the scientific community. The studies involved had the consent and voluntary participation of observed students.

### Theoretical-methodological framework

Scoping reviews use the method of mapping, summarizing, and synthesizing existing knowledge on a given topic to provide a comprehensive and systematic view of available evidence. When applied to a study involving human beings, they aim to provide an understanding of trends and gaps related to the topic^(5)^. Therefore, this study seeks to collect the various types of evidence related to undergraduate students’ mental health during the first year of the pandemic and show how they were produced.

### Study design

This is a scoping review with a qualitative approach, which allows for better exploration and understanding of the complexity of a topic. Qualitative analysis also involves an inductive approach, through which researchers seek to identify patterns in the data collected, which can be done through categorization and interpretation^(5)^.

### Methodological procedures

Inclusion and exclusion criteria were established to ensure the relevance and consistency of the studies to be reviewed. Eligibility criteria included undergraduate students, attending public or private educational institutions, of all courses, worldwide. Studies selected for the review should address undergraduate students’ mental health in the first year of the pandemic and use empirical methodologies. On the other hand, articles that addressed the mental health of elementary or high school students, students in graduate courses or vocational training, and studies that focused exclusively on therapeutic or pharmacological interventions were eliminated. These criteria were defined to ensure that the studies selected for the review were relevant to the research question and met certain methodological standards.

Furthermore, the Preferred Reporting Items for Systematic Reviews and Meta-analysis extension for Scoping Reviews (PRISMA-ScR) protocol for scoping reviews and the protocol checklist published by the EQUATOR network (Enhancing the QUAlity and Transparency Of health Research) were used.

### Study setting

Since this study covers the first year of the pandemic, empirical articles published in 2020 from any country involving human participants and describing intervention proposals for these students’ mental health problems were included. The emphasis given to the first year of the pandemic – which can be said to have been more significant since March 2020 – is due to the intention of identifying the ways of dealing with and solutions sought in different contexts and countries, given the unprecedented global challenges of the COVID-19 pandemic.

### Data collection and organization

To map articles relevant to this review, searches were carried out in the following bibliographic databases: SciELO, VHL (LILACS), EBSCO, PubMed (MEDLINE) and CAPES journals (Scopus, Web of Science, Embase). The article search process took place between July 2022 and January 2023. The search was carried out by descriptors, using Boolean operators in the combination of the terms “COVID Pandemic”; “Mental Health”, “Students”, also associated with the descriptors “Spirituality” OR “Religiosity” OR “Existential Analysis”, “Meaning of Life”. A data mapping form was developed to determine the variables to be extracted from the studies found. The form allowed to collect information on study design, sample and country, objectives, main findings, topics, and proposed interventions.

Studies were grouped by country of origin, and findings were described both narratively and diagrammatically.

## RESULTS

**Figure 1:**
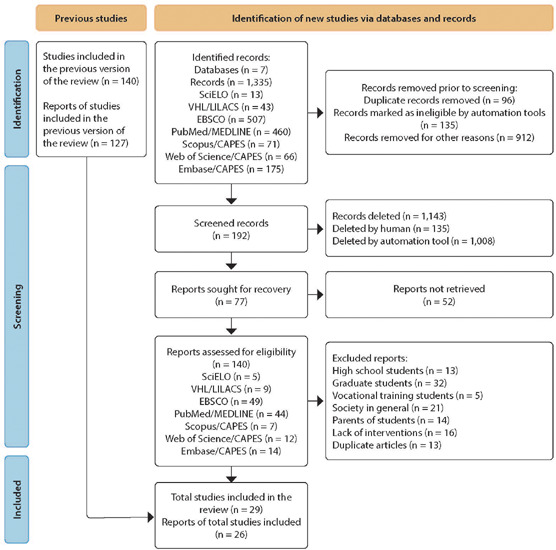
Articles selected for scoping review

*Source: PRISMA, 2020.*

The search for articles found a total of 1,335 studies within the established databases and descriptors. After applying the eligibility criteria, 140 articles that fit the conceptual framework of this scoping review were selected. Exclusion by type of content and participant involved was applied, in addition to excluding duplicate articles found in different databases. Thus, for the final selection, 26 articles were included, as shown in the PRISMA 2020 flowchart below (Figure 1).

Chart 1 describes the characteristics (author, type of content, country of origin and objectives) of the articles selected for this scoping review as well as the participants involved. All selected articles are from 2020.

**Chart 1:** Characteristics of the articles and participants involved, Niterói, Rio de Janeiro, Brazil, 2022

Country of origin and author	Study design	Objectives	Participants
United States of America (Son *et al.*)^(2)^	Qualitative, quantitative	Carry out an assessment of the effects of the COVID-19 pandemic on undergraduate students’ mental health.	195 students
France (Essadek, Rabevron)^(4)^	Quantitative, online questionnaires, three scales	Assess the impact of COVID-19 on French students’ mental health in eastern France, which was the first and most affected region by the pandemic.	8,004 students
Bangladesh (Ahmmed, Maria)^(6)^	Descriptive, scales and questionnaires	Explore the factors that influence the level of anxiety of students graduating during this pandemic.	52 students
China (Ma *et al.*)^(7)^	Cross-sectional	Assess mental health issues and associated factors in students.	746,2,7 students
United States of America (Ruiz *et al.*)^(8)^	Descriptive, logistic regression analysis	Explore the relationship between life disruptions due to COVID-19 and the severity of anxiety.	353 students
China (Chen *et al.*)^(9)^	Cross-sectional observational	Determine students’ rhythms of life and mental health during quarantine.	323,489 students
Turkey (Aslan, Ochnik, Çιnar)^(10)^	Qualitative, scales, questionnaires	Reveal the prevalence of perceived stress and mental health among students during the pandemic and explore predictors of stress levels.	358 students
Germany (Schlichtiger *et al.*)^(11)^	Cohort (longitudinal), qualitative	Explore the psychological effects of the COVID-19 crisis on a sample of students from Bavaria.	1,106 students
Peru (Saravia Bartra *et al.*)^(12)^	Descriptive, scales, analysis	Determine the level of anxiety present in first-year medical students at a private university in Lima, Peru.	57 students
Bangladesh (Khan *et al.*)^(13)^	Cross-sectional, quantitative	Assess the level of psychological impact on undergraduate students who have to quarantine.	505 students
United States of America (Kleiman *et al.*)^(14)^	Descriptive, ecological momentary assessment	Assess the nature of the impact on the mental health of undergraduate students who were experiencing the pandemic in real time.	140 students
Saudi Arabia (Meo *et al.*)^(15)^	Quantitative, online questionnaire	Investigate the impact of quarantine on medical students’ mental well-being.	625 medical students
Spain (Ferro *et al.*)^(16)^	Descriptive, non-experimental cross-sectional	Describe the psychological impact of COVID-19 on medical students.	63 students
Brazil (Miskulin *et al.*)^(17)^	Prospective study, scales	Compare the prevalence of depressive symptoms during the COVID-19 pandemic quarantine in undergraduate students and explore possible factors related to this.	347 students
Brazil (Pereira *et al.*)^(18)^	Prospective study, questionnaires	Prospectively assess the prevalence of common mental disorders (CMDs) in medical students before and during the COVID-19 quarantine over a three-year period.	860 medical students
United States of America (Gonzales *et al.*)^(19)^	Quantitative, qualitative, online questionnaire	Examine LGBT undergraduate students’ mental health needs in the US during the COVID-19 pandemic.	477 LGBT students
China (Chi *et al.*)^(20)^	Online cross-sectional survey	Investigate the prevalence and risk factors for student mental health issues during the pandemic.	2,038 students
Japan (Arima *et al.*)^(21)^	Cross-sectional, quantitative, online questionnaire with scales	Assess factors associated with psychological distress among medical students during the period of forced home quarantine from March to May 2020.	57, medical students
Turkey (Arslan *et al.*)^(22)^	Descriptive, multi-group	Examine the effect of meaning in life on overall mental health, which represents the presence of positive functioning and the absence of psychopathological symptoms.	392 students
Indonesia (Fitriyah *et al.*)^(23)^	Qualitative, online surveys	Explore the effect of spirituality on students during the COVID-19 pandemic.	1,004 students from 65 universities
China (Yu, Yu, Li)^(24)^	Qualitative, scales and questionnaires	Pay attention to students’ mental health and its link to meaning in life.	932 students
Iraq (Yildirim, Arslan, Aziz)^(25)^	Qualitative, scales and questionnaires	Test the mediating roles of resilience and meaning in life between COVID-19 worry and mental health disorders.	284 students
Ukraine (Rogowska *et al.*)^(26)^	Cross-sectional, online survey	Examine the relationship between physical activity (PA) and mental health among Ukrainian undergraduate students during the COVID-19 pandemic lockdown.	1,512 students
Italy (Amatori *et al.*)^(27)^	Observational, descriptive	Investigate the effects of mood states and exercise on nutritional choices in undergraduate students during the pandemic.	176 students
United States of America (Scharmer *et al.*)^(28)^	Descriptive, online questionnaire	Examine associations between COVID-19 anxiety, intolerance and pathology of erectile dysfunction (ED) and compulsive exercise.	295 students
United Arab Emirates (Drissi *et al.*)^(29)^	Quantitative, online questionnaire	Assess the psychological effects of the COVID-19 lockdown on undergraduate students.	154 students

Chart 2 describes article results (main findings and proposed interventions).

**Chart 2:** Main results and intervention proposals found, Niterói, Rio de Janeiro, Brazil, 2022

Country of origin and author	Main findings	Proposed interventions
United States of America (Son *et al.*)^(2)^	Of the 195 students, 138 (71%) indicated increased stress and anxiety due to the COVID-19 outbreak. Stressors identified included fear and concern for their own health and that of loved ones (177/195, 91%), difficulty concentrating (173/195, 89%), disruptions to sleep patterns (168/195, 86%), decreased social interactions due to physical distancing (167/195, 86%), and increased concern about academic performance (159/195, 82%).	Educate and inform students about the possibilities of accessing online mental healthcare services offered inside and outside the university.
France (Essadek, Rabevron)^(4)^	When surveying 8,004 students, it was observed that they suffer from particularly high levels of anxiety, depression and distress. A significant proportion of students may require psychological support.	Identify and promote psychological counseling for students at greater risk of developing post-traumatic stress symptoms.
Bangladesh (Ahmmed, Maria)^(6)^	Of the 52 students, 13.46% had severe anxiety. In addition, living in an urban area, being poor, and having relatives or acquaintances infected with COVID-19 were risk factors for anxiety.	Monitor students’ mental health to provide crisis-oriented mental healthcare services to alleviate anxiety for those who need it most.
China (Ma *et al.*)^(7)^	Of the 746,217 students, approximately 45% had mental health problems. The prevalence of probable acute stress, depressive and anxiety symptoms was 34.9%, 21.1% and 11.0%, respectively. The COVID-19 epidemic factor that was associated with an increased risk of mental health problems was infection of relatives or friends.	Provide psychosocial support and services to students most at risk for psychological distress due to risk factors.
United States of America (Ruiz *et al.*)^(8)^	Across the 353 students, specific disruptions, such as difficulty accessing basic necessities, difficulty working from home, and difficulty transitioning to remote learning, were found to increase the likelihood of experiencing clinical levels of anxiety. These disruptions led 13 students to attempt life-disruption techniques.	Counseling so students know how to access basic needs and promote enhanced remote learning for everyone to access.
China (Chen *et al.*)^(9)^	Of the 323,489 students, 7.7% experienced depressive symptoms during the COVID-19 pandemic. These students had low emotional regulatory self-efficacy. Browsing information about COVID-19 for more than three hours per day was associated with depressive symptoms. Unfavorable life rhythms were associated with depressive symptoms.	Provide psychological support to prevent mental health problems in undergraduate students during the COVID-19 pandemic.
Turkey (Aslan, Ochnik, Çιnar)^(10)^	Among the 358 students, there was high perceived stress, mild generalized anxiety, and low life satisfaction. More than half of students met diagnostic criteria for generalized anxiety disorder (52%) and depression (63%).	Provide online psychological counseling services.
Germany (Schlichtiger *et al.*)^(11)^	Of the 1,106 students, 17.3% (n=336) indicated that they experienced less mental stress during the COVID-19 pandemic, whereas 39.6% (n=770) reported an increased psychological burden.	Explore mental state changes in young adults during the pandemic lockdown and provide necessary psychological services.
Peru (Saravia Bartra *et al.*)^(12)^	Of the 57 students, 75.4% reported some degree of anxiety. A statistically significant association was found between being female and anxiety (p = 0.045). Similarly, there was no association between age and anxiety (p = 0.058).	Offer psychological support services in university centers.
Bangladesh (Khan *et al.*)^(13)^	Of the 505 students, 28.5% presented stress, 33.3%, anxiety, 46.92%, mild to extremely severe depression, and 69.31% had mild to severe event-specific distress.	Implement mental health programs by the authorities, including multidimensional psychological interventions.
United States of America (Kleiman *et al.*)^(14)^	Among the 140 students, 15% reported high levels of anxiety due to COVID-19 every day. Participants felt more anxious on days when the number of new cases and deaths due to COVID-19 was higher.	Assist in addressing mental health problems. Provide psychological support to reduce anxiety and sadness and rehabilitation for students who use alcohol and drugs.
Saudi Arabia (Meo *et al.*)^(15)^	Of the 625 students, a combined cohort of 234 female and male medical students (44.1% of the total respondents) showed a sense of being emotionally distant from family, friends, and colleagues. Students showed a marked decrease in their overall work performance. In addition to this, 56.2% of the total students (61.5% female and 49.5% male) felt a decrease in study time.	Offer psychological care to all students while they are in quarantine to improve mental well-being.
Spain (Ferro *et al.*)^(16)^	Among the 63 students, it was observed that 33.3% maintained normal levels of stress, whereas 42.9% presented stress, 19%, excessive levels, and 4.8%, excessive stress, which means that 66.7% presented stress levels.	Work on spirituality in order to promote the mental health and emotional well-being of students who seek care.
Brazil (Miskulin *et al.*)^(17)^	Among the 347 students, the Hospital Anxiety and Depression Scale (HADS-D) >8 (above the cut-off point) had a prevalence of 36% (n=125). First-year students had a higher prevalence of HADS-D >8 (45.6%) compared to other years (32%) (p=0.015).	Offer psychotherapeutic care to all students who seek it at the university.
Brazil (Pereira *et al.*)^(18)^	Among the 860 students, the frequency above the cut-off point for CMD was 60.5%, and the mean scores were 8.2±4.6. No difference was found in the sample in the years 2018, 2019, and 2020 for either CMD (62.2%, 60.9%, and 59.2%, respectively; p=0.762) or scores (p=0.351).	The prevalence of CMD in these students did not change during the pandemic; therefore, no intervention proposals were presented.
United States of America (Gonzales *et al.*)^(19)^	Of the 477 LGBT students, nearly half (45.7%) have immediate families who are not supportive or aware of their LGBT identity. Approximately 60% of LGBT students in the sample were experiencing psychological distress, anxiety, and depression during the pandemic.	Offer psychological support for mental health and reduce LGBT students’ psychological distress during the pandemic.
China (Chi *et al.*)^(20)^	Among the 2,038 students, the prevalence of clinically relevant post-traumatic stress disorder, anxiety and depressive symptoms, and post-traumatic growth was 30.8%, 15.5%, 23.3%, and 66.9%, respectively.	Offer psychological support and work on students’ resilience, even remotely.
Japan (Arima *et al.*)^(21)^	Of the 571 students, 163 (28.5%) indicated a significant degree of psychological distress.	Offer psychological care aimed at increasing self-esteem and self-efficacy, with a focus on improving personal resilience.
Turkey (Arslan *et al.*)^(22)^	When observing 392 students, it was found that the analyses of latent variable trajectories demonstrated significant trajectories of meaning in life for all components of psychological distress, positive mental health and subjective well-being.	Counseling to help students find meaning in life and use this to promote full psychological functioning.
Indonesia (Fitriyah *et al.*)^(23)^	For the 1,004 students, the Smart-PLS application was used to analyze statistical data, and it was observed that spirituality and empathy have a significant reciprocal relationship (with a path coefficient of 0.564). Spirituality predicts empathy and vice versa.	Apply spiritual strategies in student counseling activities to improve their empathy and anticipate future psychological crises.
China (Yu, Yu, Li)^(24)^	Among the 932 students, mindfulness was associated with lower levels of depression, anxiety, and stress. Meaning in life factors were negatively related to depression, anxiety, and stress (all Ps < .01). Further analyses suggested that indirect effects accounted for 56.52% of the total effect of mindfulness on depression, 55.56% of the total effect of mindfulness on anxiety, and 47.83% of the total effect of mindfulness on stress.	Provide mindfulness-based psychological care and counseling to improve the level of meaning in life and help alleviate psychological distress during the COVID-19 pandemic.
Iraq (Yildirim, Arslan, Aziz)^(25)^	Across 284 participants, worry about COVID-19 negatively predicted resilience and meaning in life and positively predicted mental health disorders.	Offer psychological support to reduce the impacts of specific pandemic stressors on mental health.
Ukraine (Rogowska *et al.*)^(26)^	Of the 1,512 students, 43% engaged in PA ≥150 minutes per week; 24% met criteria for generalized anxiety disorder; and 32% met criteria for depression. More students were engaged in PA before the COVID-19 outbreak than during the national lockdown. Students with anxiety and depression were almost twice as likely to engage in PA as their peers without mental health disorders.	Introduce online cardiovascular PA training on e-learning platforms, led by a professional sports coach.
Italy (Amatori *et al.*)^(27)^	Among the ,76 students, exercise was positively associated with the consumption of fruits, vegetables and fish. Depression and quality of life were associated with the consumption of cereals, legumes and low-fat meats. Exercise mediated the effect of mood states on the consumption of fruits, vegetables and fish, respectively, accounting for 4.2% and ,.8% of the total variance.	Encourage cardiovascular exercise and healthier nutritional choices.
United States of America (Scharmer *et al.*)^(28)^	Among the 295 students, COVID-19 anxiety was more strongly related to compulsive exercise and ED pathology for individuals with lower intolerance of uncertainty.	Provide psychological support to students with lower levels of mental health to manage their psychological distress.
United Arab Emirates (Drissi *et al.*)^(29)^	The ,54 students were experiencing psychological problems related to depression and anxiety as well as social dysfunction. The results also revealed a lack of awareness about mental health apps and uncertainty about using such apps.	Improve students’ digital health literacy by increasing their awareness of mental health apps and their treatment methods and benefits.

The samples of empirical studies ranged from a minimum of 52 students from a university in Bangladesh^(6)^ to a maximum of 746,217 in a study of Chinese students^(7)^. In total, the samples are from 1,089,922 undergraduate students globally. Students were from various undergraduate courses, but the most frequent studies focused on students in the health field.

Studies were obtained from 16 countries. The United States of America presents the most studies on undergraduate students’ mental health, with a total of five (19.25%), followed by China, with four (15.4%) studies. Brazil, Bangladesh and Turkey present two (7.7%) studies each. The other countries, such as Germany, Saudi Arabia, United Arab Emirates, Spain, France, Indonesia, Iraq, Italy, Japan, Peru and Ukraine, present one (3.85%) study each. Regarding the methodology of each study, there are nine (34.65%) qualitative, six (23.1%) quantitative, eight (30.8%) descriptive and three (11.55%) prospective/observational studies.

Through comprehensive analysis of the 26 selected articles, it was possible to identify that the main focuses were organized into topics and subtopics, as narratively summarized below.

### Undergraduate students’ mental health

This subtopic consists of 14 articles^(2, 4, 8, 9, 10, 11, 12, 13, 14, 15, 16, 17, 18, 19)^. The studies analyze undergraduate students’ mental health during the pandemic period and present intervention proposals for psychological issues resulting from the disease. In total, 336,579 students were observed.

Of the total number of studies, five found that among the students observed, there was a prevalence of moderate to severe levels of anxiety and depression as a result of the COVID-19 pandemic. Higher levels of anxiety and depression are associated with decreased mental health and higher rates of suicidal thoughts during the pandemic. Furthermore, levels of anxiety and depression increased during the pandemic, and mental well-being decreased, precisely due to isolation and students’ inability to deal with stressful situations^(8, 9)^. Stress levels were also significantly elevated, contributing to low life satisfaction^(10)^. Some authors^(9)^ found that students who spend more than three hours a day browsing information about COVID-19 are also predisposed to increased levels of anxiety. On the other hand, other authors^(11)^ found that some undergraduate students’ mental stress during the COVID-19 pandemic decreased, although they showed an increase in psychological burden. Anxiety and stress are more present in females, and individuals with high altruism are able to balance the negative levels of these conditions^(10, 12)^.

The aforementioned authors advocate an intervention focused on reducing anxiety and depression levels through activities that allow interaction and remote psychological support for students, which enable them to continue carrying out PA and which promote quality education, satisfaction of basic needs and self-care actions.

Furthermore, six articles concluded that the COVID-19 pandemic had impacts on other health conditions and quality of life, which contributed to declining undergraduate students’ mental health. Concerns about health and academic performance, fear of infection and inadequate food supply, difficulty concentrating, interruptions in sleep patterns, decreases in social interactions, and the absence of physical and recreational activities are identified as negative psychological effects resulting from the pandemic^(2, 13)^. With the pandemic, levels of anxiety, depression, stress, anguish, sadness, worry, and panic accentuated, directly impacting students’ mental health^(4, 4)^. Furthermore, the pandemic affected other health conditions and quality of life, such as sleep quality, well-being, and sex life, which became worse, and students became more discouraged, prone to suicidal and self-destructive thoughts, with a chance of developing post-traumatic stress^(4, 5)^.

It was found that students who perceive the pandemic as an opportunity to grow in their careers and work independently have medium to high levels of optimism, proactivity, satisfaction of psychological needs and entrepreneurial intention, which contributes to reducing mental health problems^(16)^. In general, the aforementioned authors advocate preventive interventions and strategies addressing mental health, focusing on improving quality of life and promoting emotional well-being through psychological support, PA and adequate sleep, in addition to university support to ensure that academic goals are achieved.

Hence, two articles addressed undergraduate students’ mental health before and during the COVID-19 pandemic, identifying that there was a significant increase in associated stressors and a decrease in mental health. In one article^(17)^, significantly higher levels of depression, anxiety, and stress were identified in undergraduate students during the pandemic period compared to the period before the pandemic. In the other article^(28)^, no significantly altered levels of mental disorders were found in students when observed in 20,8, 20,9, and 2020. Students who lived alone or with roommates had these conditions more pronounced than students who lived with their parents and who had access to an indoor space with a yard and garden. Although some authors^(18)^ did not identify significant changes, others^(17)^ suggest that each student be monitored individually to identify the psychotherapeutic needs that they may present and that the university promote psychological care according to each need.

It should be added that an article presented data indicating that COVID-19 contributed to several students’ psychological distress. LGBT undergraduate students suffered great psychological impacts during the pandemic period because many already face anxiety and depression due to the lack of acceptance and support of their identity by their families, conditions that have worsened with the pandemic^(19)^. The authors advocate interventions with psychological support during the pandemic for mental health^(19)^.

### Stressors and factors associated with mental health problems

This category includes four studies^(6, 7, 20, 21)^, with a total sample of 748,878 undergraduate students. Of these, three articles presented the most common factors found among undergraduate students that contribute to increased risk of mental health problems during the pandemic. Students with more time in college, located in rural or peripheral areas, with a history of drinking alcohol, low social support, with infected relatives or acquaintances, and female students are more likely to develop mental health problems as a result of the pandemic^(6, 7)^. Mild anxiety and depression are among the first conditions to appear in these students as well as acute stress^(7)^. The most common mental health problems among undergraduate students are first anxiety, followed by depression and stress. Issues related to academic life – uncertainties about the academic program and personal fulfillment, changes in the teaching format –, related to health – personal health, that of friends and family –, related to the availability of reliable information about COVID-19 and related to the lack of social support are also factors considered^(20)^. The authors advocate psychological support with a focus on mental well-being as interventions.

Another study^(21)^ found that psychological distress is a consequence that can arise in a large percentage of students who develop mental health problems. Therefore, they suggest that universities should support mental health and educational initiatives aimed at increasing self-esteem and self-efficacy.

### Impact of spirituality and meaning in life on students’ mental health

Four studies^(22, 23, 24, 25)^ included in this category stand out, which investigated the relationship between the meaning of life during the COVID-19 pandemic and mental health in 2,612 undergraduate students. Of these, two articles identified positive effects of maintaining a meaning in life for improving mental health and well-being. Students who traced meaningful trajectories of meaning of life during the pandemic period, maintaining their spirituality, were better able to cope with psychological distress and maintain their mental health^(22, 23)^. Furthermore, spirituality was associated with empathy^(23)^. The authors therefore suggest approaches and counseling focused on meaning of life and spiritual strategies as interventions for mental health problems.

According to two articles, there is a close relationship between meaning in life and mental health. The presence of meaning in life is associated with lower levels of depression, anxiety, and stress^(24)^. In addition to this observation, concern about COVID-19 has negative effects on meaning in life and resilience among undergraduate students, in addition to contributing to the onset of mental health disorders^(25)^. Therefore, the authors suggest intervention programs focusing on meaning in life, mindfulness, and resilience, which are strengths for reducing stressors and improving mental health.

### Relationship between physical activity and mental health

This topic was discussed in three studies^(26, 27, 28)^, comprising 1,983 undergraduate students, who were investigated about PA during the pandemic and its impact on these individuals’ mental health and well-being.

Two articles concluded that regular exercise contributes to reducing anxiety, depression, and stress levels and, consequently, improving mental health, since the body releases hormones that lead to a feeling of well-being. Some authors^(26)^ analyzed a physically active group and an inactive group of students and observed that the inactive group had higher anxiety and depression scores, and identified that stress increased in students who spent the first weeks of isolation without practicing PA. Others^(27)^ noticed that exercise contributes to positive mood states in undergraduate students, in addition to being associated with the consumption of fruits, vegetables, and fish. On the other hand, depression and low levels of mental health were associated with the consumption of cereals, legumes, and low-fat meats, in addition to a sedentary lifestyle. The authors advocate PA and healthy eating to improve students’ mental health levels.

It was also identified that anxiety caused by COVID-19 was strongly related to compulsive exercise as well as ED, and this was more common in students with lower intolerance to uncertainty^(28)^. Thus, the authors suggest prevention and intervention efforts aimed at intolerance, uncertainty and anxiety, in addition to interventions aimed at managing psychological distress^(26, 27, 28)^.

### Mental health and digital education

In a study^(29)^ included in this category, 154 undergraduate students were observed to understand how the replacement of in-person teaching with remote teaching due to the pandemic impacted their mental health. The research^(29)^ investigated students’ attitude regarding the use of digital applications and identified that many felt insecure and uncertain about their use due to a lack of awareness and knowledge about applications. Thus, the authors suggested improving students’ literacy in digital health, increasing their awareness and presenting them with the benefits of applications.

## DISCUSSION

Through these studies, the prevalence of anxiety, depression, and stress among students due to the pandemic was discovered, contributing to increased risk of developing mental health problems. Initially, it was found that the development of psychological problems and disorders resulting from the pandemic is specific to each student and their different conditions. Some authors^(18)^ concluded that the pandemic had minimal effects on students’ mental health. Some^(11)^ noticed that, instead of an increase or no change in mental health problems, there was a reduction in mental stress among some undergraduate students during the pandemic. Others^(24, 25)^ also observed minimal effects on the mental health of students who maintained a meaning for life and their spirituality during the pandemic, with, at most, mild levels of depression observed in a few students.

On the other hand, other authors^(10, 17, 20)^ found that the pandemic significantly increased the levels of anxiety, depression, and stress in these individuals, leading them to develop mental health disorders, the onset of mental disorders, and decreased well-being. Others^(4, 14)^ point out that, in addition to anxiety, depression, and stress, mental health conditions most frequently found in students as a result of the pandemic, anguish, sadness, concern about life and academic development, fear, and panic had their levels accentuated, also contributing to reduced mental health.

Most studies have found that mental health issues are more common in women. They have concluded that anxiety, depression, and stress are more common among female students^(6, 7, 10, 12, 16, 17)^.

One of the risk factors observed, related to increased mental health problems, is spending some hours a day browsing information and gaining more knowledge about COVID-19, which leads to high levels of anxiety^(9)^. Certain studies^(2, 6, 7, 13, 26, 29)^ also point out as risk factors the concern for one’s own health and family’s and friends’ health, fear of infection, fear of lack of food, concern about academic performance, difficulty concentrating, interruptions in sleep patterns, decreases in social interactions, lack of PA and recreational activities, living alone or with roommates, having more time in school, living in rural or peripheral areas, having a history of drinking alcohol, having low social support, changes in the teaching format, having financial and technical difficulties in using digital platforms, suffering isolation on social networks, insecurity and uncertainty in using teaching applications during the pandemic period, inactivity, and a sedentary lifestyle.

High levels of anxiety, depression and stress are associated with higher rates of suicidal and self-destructive thoughts during the pandemic, as well as poor quality of sleep, well-being and sex life, causing discouragement and increasing the chances of students developing post-traumatic stress^(4, 8, 15)^. Mental health problems can also lead to psychological distress and other alarming psychological problems, in addition to leading to the need to exercise compulsively and ED^(19, 21, 28)^.

Given the consequences of the COVID-19 pandemic on undergraduate students’ mental health, some studies have focused on presenting strategies for coping with and overcoming risk factors associated with increased anxiety, depression, and stress. PA has been identified as one of the best practices for maintaining mental health during COVID-1 9^(26, 27)^. The authors observed that exercise contributes to lower levels of anxiety, depression, and stress scores, helps mental well-being, improves mood, and is associated with a healthier diet, which is also an important factor for mental health^(26, 27)^. Other authors^(22, 23)^ argue that spirituality is also a factor that contributes to mental health balance, as it allows students to find meaning and purpose in life, even amid the chaos of a pandemic. They also advocate the importance of acceptance, self-distraction, and active coping as coping strategies. In a general context, proposals for interventions against psychological impairment were observed in the articles discussed, highlighting, mainly, strategies for psychological and emotional support for students.

Studies that investigated a larger number of undergraduate students, with over 500 participants, for instance, provided more specific data on the consequences of the COVID-19 pandemic on their mental health, demonstrating that, in fact, there was harm to a considerable portion of students worldwide, who presented high levels of anxiety, depression and stress. These same data were not observed in several of the studies that investigated a smaller number of students, with less than 100, for instance. In these studies, the data indicated few psychological changes and minimal consequences for mental health. It is necessary to consider, however, the difficulty of obtaining more accurate data with few participants in a study, since often socioeconomic conditions among them may not differ significantly. The same does not occur with studies involving thousands of participants, since the chances of them having wide socioeconomic, experience and health differences are greater, and this impacts study results. Furthermore, it is important to consider the differences among countries, since this is a study of a global context. Research with students in poorer countries with fewer conditions to deal with the pandemic tends to present different results from research carried out with students in countries and universities that are better prepared for the current situation.

This scoping review found that there is considerable evidence on the issue in national and international literature, but the main focus of these studies was on Chinese undergraduate students, who were investigated in greater numbers. This may suggest that a future systematic review may be appropriate for investigating undergraduate students’ mental health in China. However, there is a lack of more complete evidence related to other countries, which implies the need for more specific data, and does not indicate the need for systematic reviews for the content.

### Study limitations

This scoping review is restricted to the search for evidence only, so the selection of included studies was subject to bias from specific inclusion and exclusion criteria, as well as to the subjective interpretation of researchers.

### Contributions to health and public policies

This review provides a deeper understanding of the factors that have impacted undergraduate students’ mental health during the COVID-19 pandemic, which may provide insights for developing targeted intervention and support strategies. It also provides insights into effective interventions that can be implemented to mitigate the negative impacts of the pandemic.

The study can also contribute to the work of mental healthcare professionals, educators and policy makers, since, by highlighting the challenges faced by undergraduate students and possible interventions, it guides decision-making and the development of a support program appropriate to the specific needs of this population.

## FINAL CONSIDERATIONS

The scoping review presented here highlights the relevance of studying undergraduate students’ mental health, highlighting the urgent need to develop care and protection strategies in this area. The COVID-19 pandemic has significantly increased the stressors faced by these students, exacerbating mental well-being issues. This increase in stressors is reflected in symptoms such as anxiety, depression and feelings of isolation, which have become more prevalent during the pandemic.

As coping strategies, it is suggested that incorporating regular PA, healthy eating, practicing spirituality, and finding meaning in life can serve as important anchors for many students, offering a path to finding peace and purpose even during difficult times. Psychological and emotional support, whether through professional counseling or social support networks, is also crucial in helping students navigate these challenging experiences.

While existing literature already provides relevant data for discussion, there is still a lack of country-specific information, which prevents a full understanding of the problem on a global scale. This gap suggests the need for more comprehensive and detailed research that can capture the nuances of students’ experiences in different cultural and social contexts.

Undergraduate students’ mental health has been severely impacted by the pandemic, and the creation of policies and programs to support mental health is crucial. Educational institutions must actively engage in the implementation of mental healthcare services, offering appropriate and accessible support to their students. Furthermore, there is a need for continued awareness of the importance of mental health, not only in times of crisis, but as a comprehensive part of student well-being in general. Prioritizing mental health is essential to ensure that students can face academic and personal challenges with resilience and adequate support.
