# Supplementary material for: Undergraduate students’ mental health in the first year of the COVID-19 pandemic: a scoping review
Source: Rev Bras Enferm. 2025 Mar 10;78(1):e20240032. doi: 10.1590/0034-7167-2024-0032 (PMC11893065; doi:10.1590/0034-7167-2024-0032)
Supplement: Translation

# Translation: Saúde mental do estudante universitário no primeiro ano da pandemia de COVID-19: revisão de escopo

**DOI:** 10.1590/0034-7167-2024-0032pt

**Authors:** Fabio Araujo Dias, Eliane Ramos Pereira, Rose Mary Costa Rosa Andrade Silva, Angelica Yolanda Bueno Bejarano Vale de Medeiros, Janaina Mengal Gomes Fabri

**Affiliations:** I Universidade Federal Fluminense. Niterói, Rio de Janeiro, Brasil

**Keywords:** Saúde Mental, Pandemia, COVID-19, Estudantes, Saúde

## Abstract

**Objetivos:**

mapear as evidências da saúde mental de estudantes de graduação em um contexto global durante a pandemia de COVID-19.

**Métodos:**

revisão de escopo, na qual o PRISMA-ScR foi utilizado.

**Resultados:**

incluíram-se 26 artigos, dos quais foram coletados dados sobre as características dos artigos, participantes envolvidos e resultados. Os resultados foram categorizados em: Estado de saúde mental dos universitários; Estressores e fatores associados aos problemas de saúde mental; Impacto da espiritualidade e do sentido da vida na saúde mental dos estudantes; Relação entre atividade física e saúde mental; Saúde mental e ensino digital.

**Considerações Finais:**

a pandemia intensificou os desafios de saúde mental enfrentados pelos estudantes universitários, evidenciando a necessidade de intervenções estratégicas. Sugere-se que instituições de ensino implementem programas de suporte psicológico, incentivem práticas saudáveis, espiritualidade e busca de sentido. Percebe-se que tais medidas podem mitigar os efeitos negativos da pandemia e fortalecer a resiliência dos estudantes.

## INTRODUÇÃO

No mês de dezembro de 2019, em Wuhan, província de Hubei, China, ocorreram os primeiros relatos do surgimento de uma nova pneumonia causada pelo novo coronavírus (SARS-CoV-2), dando origem à doença que foi denominada COVID-19^(1)^. Autores^(2)^ apontam que a prevalência de epidemias acentua e cria novos estressores. Além do medo, preocupação e tristeza diante de uma crise sanitária dessa amplitude, a população enfrenta restrições ao movimento físico e atividades sociais, bem como mudanças radiais e repentinas no estilo de vida, impactando a saúde mental dos indivíduos, sua espiritualidade e o significado da vida^(3)^.

A pandemia afetou, de formas diferentes, populações específicas. Assim, citam-se, aqui, os estudantes universitários, que são, também, conhecidos como uma população vulnerável nesse período pandêmico e sobre a qual ainda há carência de estudos relacionados aos efeitos psicológicos da doença na sua saúde mental^(4)^. Pesquisas indicam que os estudantes de graduação, muitas vezes, já enfrentam questões psicológicas, como ansiedade, pânico e depressão, devido às complicações dos cursos de nível superior. Com a pandemia, novas complicações surgem. São instituições de ensino fechadas, semestres perdidos, teses adiadas, substituição do ensino presencial para o ensino remoto e mais^(3)^.

Dessa forma, foi formulada a seguinte questão: quais as evidências científicas acerca da saúde mental dos estudantes universitários no primeiro ano pandémico e as propostas de intervenção adotadas nesse período?

Espera-se contribuir com o conhecimento atual sobre a saúde mental dos universitários, enriquecendo o campo da saúde pública com dados sobre as repercussões da COVID-19 na vida e saúde mental dos estudantes universitários no primeiro ano do período pandêmico, e as intervenções aplicadas naquele contexto.

Neste trajeto, este estudo, ao identificar as necessidades dos estudantes, trazer a compreensão dos impactos da pandemia, identificar os fatores de risco e intervenções propostas e realizar uma comparação entre países, mostra-se relevante ao contribuir para que sejam orientados esforços para mitigar os impactos e melhorar a qualidade de vida desses jovens, desenvolvendo estratégias de prevenção e intervenção mais eficazes e personalizadas.

## OBJETIVOS

Mapear as evidências da saúde mental de estudantes de graduação em um contexto global durante a pandemia de COVID-19.

## MÉTODOS

### Aspectos éticos

Os estudos incluídos nesta revisão de escopo demonstram preocupação com os aspectos éticos envolvidos na pesquisa com participantes humanos. Assim, apresentaram abordagem para garantir a proteção da integridade dos participantes, seguindo as diretrizes éticas estabelecidas pela comunidade científica. Os estudos envolvidos contaram com o consentimento e a participação voluntária dos estudantes observados.

### Referencial teórico-metodológico

A revisão de escopo utiliza o método de mapeamento, resumo e sintetização de um conhecimento existente sobre determinado tópico, de forma a oferecer uma visão abrangente e sistemática das evidências disponíveis. Quando aplicada a um estudo com seres humanos, ela visa trazer a compreensão das tendências e lacunas relacionadas ao tema^(5)^. Logo, o presente estudo busca coletar os vários tipos de evidências relacionadas à saúde mental dos estudantes universitários durante o primeiro ano da pandemia e mostrar como elas foram produzidas.

### Tipo de estudo

Trata-se de revisão de escopo com abordagem qualitativa, que permite melhor exploração e compreensão sobre a complexidade de um tema. A análise qualitativa envolve, ainda, uma abordagem indutiva, por meio da qual os pesquisadores buscam identificar padrões nos dados coletados, o que pode ser feito por meio de categorização e interpretação^(5)^.

### Procedimentos metodológicos

Foram estabelecidos critérios de inclusão e exclusão para garantir a relevância e a consistência dos estudos a serem revisados. Os critérios de elegibilidade incluíram estudantes universitários de graduação, que frequentavam instituições de ensino, públicas ou privadas, de todos os cursos, em todo o mundo. Os estudos selecionados para a revisão deveriam abordar a saúde mental dos estudantes universitários no primeiro ano pandêmico e utilizar metodologias empíricas. Por outro lado, como critérios de exclusão, foram eliminados os artigos que abordassem a saúde mental de estudantes do ensino fundamental ou médio, estudantes universitários de pós-graduação ou cursos técnicos, e estudos que se concentrassem exclusivamente em intervenções terapêuticas ou farmacológicas. Esses critérios foram definidos para garantir que os estudos selecionados para a revisão fossem relevantes para a pergunta de pesquisa e atendessem a certos padrões metodológicos.

Ainda, foram utilizados o protocolo *Preferred Reporting Items for Systematic Reviews and Meta-analysis extension for Scoping Reviews* (PRISMA-ScR) para revisões de escopo e a lista de verificação do protocolo publicada pela rede EQUATOR (*Enhancing the QUAlity and Transparency Of health Research*).

### Cenário do estudo

Por se tratar de estudo referente ao primeiro ano do período pandêmico, foram incluídos artigos publicados em 2020, de qualquer país, empíricos, que envolvessem participantes humanos e que descrevessem propostas de intervenção aos problemas de saúde mental desses estudantes. A ênfase dada ao primeiro ano da pandemia – pode-se dizer que de modo mais significativo a partir de março de 2020 – dá-se pela intenção de identificar os modos de lidar e soluções buscadas em diferentes contextos e países, diante do ineditismo e desafios globais da pandemia de COVID-19.

### Coleta e organização dos dados

Para o mapeamento de artigos relevantes à presente revisão, as pesquisas foram realizadas nas seguintes bases de dados bibliográficas: SciELO, BVS (LILACS), EBSCO, PubMed (MEDLINE) e periódicos CAPES (Scopus, *Web of Science,* Embase). O processo de pesquisa dos artigos se deu entre julho de 2022 e janeiro de 2023. A busca foi realizada pelos descritores, utilizando os operadores booleanos na combinação dos termos “*COVID Pandemic”, “Mental Health”, “Students”,* sendo associados também aos descritores “*Spirituality” OR “Religiosity” OR “Existential Analysis”, “Meaning of Life”.* Um formulário de mapeamento de dados foi desenvolvido para determinar as variáveis a serem extraídas dos estudos encontrados. O formulário permitiu a coleta de informações sobre desenho do estudo, amostra e país, objetivos, principais descobertas, temas, e intervenções propostas.

Os estudos foram agrupados por país de origem, e as descobertas foram descritas de forma narrativa e diagramática.

## RESULTADOS

A busca de artigos encontrou, dentro das bases de dados e descritores estabelecidos, um total de 1.335 estudos. Aplicados os critérios de elegibilidade, foram selecionados 140 artigos que se enquadravam na estrutura conceitual desta revisão de escopo. Foi aplicada a exclusão por tipo de conteúdo e por participante envolvido, além de serem excluídos artigos duplicados encontrados em diferentes bases de dados. Assim, para a seleção final, foram incluídos 26 artigos, conforme apresenta o fluxograma PRISMA 2020 que se segue (Figura 1).

**Figura 1:** Artigos selecionados para a revisão de escopo

*Fonte: PRISMA, 2020.*

As características (autor, tipo de conteúdo, país de origem e objetivos) dos artigos selecionados para esta revisão de escopo, bem como os participantes envolvidos, estão descritos no Quadro 1. Todos os artigos selecionados são de 2020.

**Quadro 1:** Características dos artigos e participantes envolvidos, Niterói, Rio de Janeiro, Brasil, 2022

País de origem e autor	Tipo de estudo	Objetivos	Participantes
Estados Unidos da América (Son *et al.*)^(2)^	Qualitativo, quantitativo	Realizar uma avaliação dos efeitos da pandemia de COVID-19 na saúde mental de estudantes universitários.	195 estudantes
França (Essadek, Rabevron)^(4)^	Quantitativo, questionários *online,* três escalas	Avaliar o impacto da COVID-19 na saúde mental de estudantes franceses na parte leste da França, que foi a primeira e mais atingida região pela pandemia.	8.004 estudantes
Bangladesh (Ahmmed, Maria)^(6)^	Descritivo, escalas e questionários	Explorar os fatores que influenciam o nível de ansiedade dos alunos que se formam nesta pandemia.	52 estudantes
China (Ma *et al.*)^(7)^	Transversal	Avaliar os problemas de saúde mental e fatores associados em estudantes.	746.217 estudantes
Estados Unidos da América (Ruiz *et al.*)^(8)^	Descritivo, análise de regressão logística	Explorar a relação entre as interrupções na vida do COVID-19 e a gravidade da ansiedade.	353 estudantes
China (Chen *et al.*)^(9)^	Observacional transversal	Determinar os ritmos de vida e a saúde mental de estudantes durante a quarentena.	323.489 estudantes
Turquia (Asian, Ochnik, Çınar)^(^,0^)^	Qualitativo, escalas, questionários	Revelar a prevalência de estresse percebido e saúde mental entre estudantes durante a pandemia e explorar preditores de níveis de estresse.	358 estudantes
Alemanha (Schlichtiger *et al.*)^(11)^	Coorte (longitudinal), qualitativo	Explorar os efeitos psicológicos da crise da COVID-19 em uma amostra de estudantes da Baviera.	1.106 estudantes
Peru (Saravia Bartra *et al.*)^(12)^	Descritivo, escalas, análise	Determinar o grau de ansiedade presente em estudantes de medicina do primeiro ano de uma universidade privada em Lima, Peru.	57 estudantes
Bangladesh (Khan *et al.*)^(13)^	Transversal, quantitativo	Avaliar o nível de impacto psicológico de estudantes universitários que devem ficar em quarentena.	505 estudantes
Estados Unidos da América (Kleiman *et al.*)^(14)^	Descritivo, avaliação ecológica momentânea	Avaliar a natureza do impacto na saúde mental de estudantes de graduação que vivenciavam a pandemia em tempo real.	,40 estudantes
Arábia Saudita (Meo *et al.*)^(15)^	Quantitativo, questionário *online*	Investigar o impacto da quarentena no bem-estar mental dos estudantes de medicina.	625 estudantes de medicina
Espanha (Ferro *et al.*)^(16)^	Descritivo, transversal não experimental	Descrever o impacto psicológico da COVID-19 em estudantes de ciências médicas.	63 estudantes
Brasil (Miskulin *et al.*)^(17)^	Estudo prospectivo, escalas	Comparar a prevalência de sintomas depressivos durante a quarentena da pandemia de COVID-19 em estudantes de graduação e explorar possíveis fatores relacionados a isso.	347 estudantes
Brasil (Pereira *et al.*)^(18)^	Estudo prospectivo, questionários	Avaliar prospectivamente durante três anos a prevalência de transtornos mentais comuns (TMC) em estudantes de medicina antes e durante a quarentena de COVID-19.	860 estudantes de medicina
Estados Unidos da América (Gonzales *et al.*)^(19)^	Quantitativo, qualitativo, questionário *online*	Examinar as necessidades de saúde mental de estudantes universitários LGBT nos EUA durante a pandemia de COVID-19.	477 estudantes com identidade LGBT
China (Chi *et al.*)^(20)^	Inquérito transversal *online*	Investigar a prevalência e os fatores de risco para problemas de saúde mental de estudantes durante a pandemia.	2.038 estudantes
Japão (Arima *et al.*)^(21)^	Transversal, quantitativo, questionário *online* com escalas	Avaliar os fatores associados ao sofrimento psíquico entre estudantes de medicina durante o período de quarentena domiciliar forçada de março a maio de 2020.	571 estudantes de medicina
Turquia (Arslan *et al.*)^(22)^	Descritivo, multigrupo	Examinar o efeito do sentido da vida na saúde mental completa, que representa a presença de funcionamento positivo e a ausência de sintomas psicopatológicos.	392 estudantes
Indonésia (Fitriyah *et al.*)^(23)^	Qualitativo, questionários *online*	Explorar o efeito da espiritualidade nos estudantes durante a pandemia de COVID-19.	1.004 estudantes de 65 universidades
China (Yu, Yu, Li)^(24)^	Qualitativo, escalas e questionários	Prestar atenção à saúde mental dos estudantes e sua ligação com o significado na vida.	932 estudantes
Iraque (Yildirim, Arslan, Aziz)^(25)^	Qualitativo, escalas e questionários	Testar os papéis mediadores da resiliência e do significado na vida entre a preocupação com a COVID-19 e os distúrbios de saúde mental.	284 estudantes
Ucrânia (Rogowska *et al.*)^(26)^	Transversal, pesquisa *online*	Examinar a relação entre a atividade física (AF) e a saúde mental de estudantes universitários ucranianos durante o bloqueio pandémico da COVID-19.	1.512 estudantes
Itália (Amatori *et al.*)^(27)^	Observacional, descritivo	Investigar os efeitos dos estados de humor e exercícios nas escolhas nutricionais em estudantes universitários durante a pandemia.	176 estudantes
Estados Unidos da América (Scharmer *et al.*)^(28)^	Descritivo, questionário *online*	Examinar associações entre ansiedade de COVID-19, intolerância e patologia de disfunção erétil (DE) e exercício compulsivo.	295 estudantes
Emirados Árabes Unidos (Drissi *et al.*)^(29)^	Quantitativo, questionário *online*	Avaliar os efeitos psicológicos do *lockdown* devido ao surto de COVID-19 em estudantes universitários.	154 estudantes

Os resultados (principais achados e intervenções propostas) dos artigos estão descritos no Quadro 2.

**Quadro 2:** Principais resultados e propostas de intervenções encontrados, Niterói, Rio de Janeiro, Brasil, 2022

País de origem e autor	Principais achados	Intervenções propostas
Estados Unidos da América (Son *et al.*)^(2)^	Dos 195 alunos, 138 (71%) indicaram aumento de estresse e ansiedade devido ao surto de COVID-19. Estressores identificados incluíram medo e preocupação com a própria saúde e de seus entes queridos (177/195, 91%), dificuldade de concentração (173/195, 89%), interrupções nos padrões de sono (168/195, 86%), diminuição das interações sociais devido ao distanciamento físico (167/195, 86%) e aumento da preocupação com o desempenho acadêmico (159/195, 82%).	Educar e informar os estudantes sobre as possibilidades de acesso aos atendimentos *online* em saúde mental oferecidos dentro e fora da universidade.
França (Essadek, Rabevron)^(4)^	Ao investigar 8.004 estudantes, observou-se que eles sofrem de níveis particularmente altos de ansiedade, depressão e angústia. Uma proporção significativa de estudantes pode necessitar de apoio psicológico.	Identificar e promover aconselhamento psicológico aos alunos com maior risco de desenvolvimento de sintomas de estresse pós-traumático.
Bangladesh (Ahmmed, Maria)^(6)^	Dos 52 estudantes, 13,46% apresentavam ansiedade severa. Além disso, morar em área urbana, ser pobre e ter parentes ou conhecidos infectados com COVID-19 foram fatores de risco para ansiedade.	Monitorar a saúde mental dos alunos para fornecer serviços mentais orientados à crise para aliviar a ansiedade daqueles que mais necessitam.
China (Ma *et al.*)^(7)^	Dos 746.217 estudantes, cerca de 45% tinham problemas de saúde mental. As prevalências de provável estresse agudo, sintomas depressivos e ansiosos foram de 34,9%, 21,1% e 11,0%, respectivamente. O fator epidêmico da COVID-19 que foi associado ao aumento do risco de problemas de saúde mental foi a infecção de parentes ou amigos.	Fornecer apoio psicossocial e serviços aos estudantes mais sujeitos a sofrimento psicológico devido aos fatores de risco.
Estados Unidos da América (Ruiz *et al.*)^(8)^	Nos 353 estudantes, observou-se que perturbações específicas, como dificuldades para acessar necessidades básicas, dificuldades em trabalhar em casa e dificuldades na transição para o aprendizado remoto, aumentam a probabilidade de experimentar níveis clínicos de ansiedade. Essas perturbações levaram 13 estudantes a tentarem técnicas de interrupção da vida.	Aconselhamento para que os estudantes saibam como acessar as necessidades básicas e promover aprendizado remoto aprimorado para o acesso de todos.
China (Chen *et al.*)^(9)^	Dos 323.489 estudantes, 7,7% apresentaram sintomas depressivos durante a pandemia de COVID-19. Estes apresentaram baixa autoeficácia emocional regulatória. Navegar por informações sobre a COVID-19 por mais de três horas por dia foi associado a sintomas depressivos. Ritmos de vida desfavoráveis foram associados a sintomas depressivos.	Fornecer apoio psicológico para prevenir problemas de saúde mental em estudantes universitários durante a pandemia de COVID-19.
Turquia (Aslan, Ochnik, Çınar)^(10)^	Entre os 358 estudantes, houve alto estresse percebido, ansiedade generalizada leve e baixa satisfação com a vida. Mais da metade dos alunos preencheu os critérios diagnósticos de transtorno de ansiedade generalizada (52%) e depressão (63%).	Fornecer serviços de aconselhamento psicológico *online.*
Alemanha (Schlichtiger *et al.*)^(11)^	Dos 1.106 estudantes, 17. 3% (n=336) indicaram que experimentaram menos estresse mental durante a pandemia de COVID-19, enquanto que 39,6% (n=770) afirmaram ter um aumento da carga psicológica.	Explorar as alterações do estado mental em adultos jovens durante o bloqueio da pandemia e oferecer os serviços psicológicos necessários.
Peru (Saravia Bartra *et al.*)^(12)^	Dos 57 estudantes, 75,4% relataram algum grau de ansiedade. Foi encontrada associação estatisticamente significativa entre sexo feminino e ansiedade (p = 0,045). Da mesma forma, não houve associação entre as variáveis idade e ansiedade (p = 0,058).	Oferecer serviços de apoio psicológico nos centros universitários.
Bangladesh (Khan *et al.*)^(13)^	Dos 505 estudantes, 28,5% apresentaram estresse, 33,3%, ansiedade, 46,92%, depressão de leve a extremamente grave, e 69,31% tiveram sofrimento específico do evento de leve a grave.	Implantar programas em saúde mental pelas autoridades, incluindo intervenções psicológicas multidimensionais.
Estados Unidos da América (Kleiman *et al.*)^(14)^	Entre os 140 estudantes, 15% relataram altos níveis de ansiedade devido à COVID-19 todos os dias. Os participantes se sentiram mais ansiosos nos dias em que o número de novos casos e mortes devido à COVID-19 era maior.	Auxiliar no enfrentamento dos problemas de saúde mental. Fornecer apoio psicológico para reduzir ansiedade e tristeza e reabilitação para estudantes que usam álcool e drogas.
Arábia Saudita (Meo *et al.*)^(15)^	Dos 625 estudantes, coorte combinada de 234 estudantes de medicina, do sexo feminino ou masculino, (44,1% do total de respondentes) mostrou uma sensação de estar emocionalmente distante da família, amigos e colegas. Os estudantes mostraram uma diminuição acentuada em seu desempenho geral no trabalho. Além disso, 56,2% do total de alunos (61,5% do sexo feminino e 49,5% do sexo masculino) sentiram uma diminuição no tempo de estudo.	Oferecer atendimento psicológico para todos os estudantes enquanto estiverem em quarentena para melhorar o bem-estar mental.
Espanha (Ferro *et al.*)^(16)^	Entre os 63 estudantes, observou-se que 33,3% mantiveram níveis normais de estresse, enquanto que 42,9% apresentaram estresse, 19%, níveis excessivos, e 4,8%, estresse excessivo, o que significa que 66,7% apresentaram níveis de estresse.	Trabalhar a espiritualidade com o intuito de favorecer a saúde mental e o bem-estar emocional dos estudantes que buscarem atendimento.
Brasil (Miskulin *et al.*)^(17)^	Entre os 347 estudantes, a prevalência de Escala Hospitalar de Ansiedade e Depressão (HADS-D) >8 (acima do ponto de corte) foi de 36% (n=125). Os alunos do primeiro ano apresentaram maior prevalência de HADS-D >8 (45,6%) em relação aos demais anos (32%) (p=0,015).	Oferecer atendimento psicoterapêutico a todos os estudantes que o buscarem na universidade.
Brasil (Pereira *et al.*)^(18)^	Nos 860 estudantes, a frequência acima do ponto de corte para TMC foi de 60,5%, e os escores médios foram de 8,2±4,6. Não foi encontrada diferença na amostra nos anos de 2018, 2019 e 2020 tanto para TMC (62,2%, 60,9% e 59,2%, respectivamente; p=0,762) quanto para escores (p=0,351).	A prevalência de TMC nesses estudantes não mudou durante a pandemia; logo, não foram apresentadas propostas de intervenção.
Estados Unidos da América (Gonzales *et al.*)^(19)^	Dos 477 estudantes universitários LGBT, quase metade (45,7%) tem famílias imediatas que não apoiam ou não conhecem sua identidade LGBT. Aproximadamente 60% dos estudantes universitários LGBT da amostra estavam passando por sofrimento psicológico, ansiedade e depressão durante a pandemia.	Oferecer apoio psicológico para a saúde mental e redução do sofrimento psicológico de estudantes LGBT durante a pandemia.
China (Chi *et al.*)^(20)^	Nos 2.038 estudantes, a prevalência de transtorno de estresse pós-traumático clinicamente relevante, ansiedade e sintomas depressivos e crescimento pós-traumático foi de 30,8%, 15,5%, 23,3% e 66,9%, respectivamente.	Oferecer apoio psicológico e trabalhar a resiliência dos estudantes, mesmo remotamente.
Japão (Arima *et al.*)^(21)^	Dos 571 estudantes, 163 (28,5%) indicaram um grau significativo de sofrimento psicológico.	Oferecer atendimento psicológico direcionado ao aumento da autoestima e autoeficácia, com foco na melhoria da resiliência pessoal.
Turquia (Arslan *et al.*)^(22)^	Ao observar 392 estudantes, verificou-se que as análises de trajetórias de variáveis latentes demonstraram trajetórias significativas do sentido da vida para todos os componentes de sofrimento psicológico, saúde mental positiva e bem-estar subjetivo.	Aconselhamento para ajudar o estudante a encontrar um significado da vida e usar isso para promover seu funcionamento psicológico completo.
Indonésia (Fitriyah *et al.*)^(23)^	Para os 1.004 estudantes, o aplicativo *Smart-PLS* foi usado para analisar os dados estatísticos, e observou-se que espiritualidade e empatia possuem uma relação recíproca significativa (com um coeficiente de trajetória de 0,564). A espiritualidade prevê empatia e vice-versa.	Aplicar estratégias espirituais nas atividades de aconselhamento ao estudante visando melhorar sua empatia e antecipar crises psicológicas futuras.
China (Yu, Yu, Li)^(24)^	Entre os 932 estudantes, a atenção plena foi associada a níveis mais baixos de depressão, ansiedade e estresse. Os fatores de significado na vida foram negativamente relacionados à depressão, ansiedade e estresse (todos Ps < 0,01). Outras análises sugeriram que os efeitos indiretos representaram 56,52% do efeito total da atenção plena na depressão, 55,56% do efeito total da atenção plena na ansiedade e 47,83% do efeito total da atenção plena no estresse.	Fornecer atendimento e aconselhamento psicológico, baseado em atenção plena, para melhorar o nível de significado da vida e ajudar a aliviar o sofrimento psicológico durante a pandemia de COVID-19.
Iraque (Yildirim, Arslan, Aziz)^(25)^	Nos 284 participantes, a preocupação com COVID-19 predisse negativamente resiliência e significado na vida e predisse positivamente transtornos de saúde mental.	Oferecer apoio psicológico com o intuito de reduzir os impactos de estressores específicos da pandemia na saúde mental.
Ucrânia (Rogowska *et al.*)^(26)^	Dos 1.512 estudantes, 43% praticavam AF ≥ 150 minutos semanais; 24% preenchiam os critérios de transtorno de ansiedade generalizada; e 32% preenchiam os critérios de depressão. Mais alunos estavam envolvidos em AF antes do surto de COVID-19 do que durante o bloqueio nacional. Estudantes com ansiedade e depressão eram quase duas vezes menos propensos a se envolver em AF do que seus colegas sem transtornos de saúde mental.	Introduzir treino de atividades físicas cardiovasculares *online* em plataformas de *e-learning,* conduzido por um treinador desportivo profissional.
Itália (Amatori *et al.*)^(27)^	Nos 176 estudantes, o exercício foi positivamente associado ao consumo de frutas, verduras e peixes. Depressão e qualidade de vida estiveram associadas ao consumo de cereais, leguminosas e carnes com baixo teor de gordura. O exercício mediou o efeito dos estados de humor no consumo de frutas, vegetais e peixe, respectivamente, respondendo por 4,2% e 1,8% da variância total.	Incentivar a prática de exercícios cardiovasculares e escolhas nutricionais mais saudáveis.
Estados Unidos da América (Scharmer *et al.*)^(28)^	Entre os 295 estudantes, a ansiedade por COVID-19 foi mais fortemente relacionada ao exercício compulsivo e à patologia de DE para indivíduos com menor intolerância à incerteza.	Oferecer atendimento psicológico aos estudantes com níveis mais baixos de saúde mental visando gerenciar seu sofrimento psicológico.
Emirados Árabes Unidos (Drissi *et al.*)^(29)^	Os 154 estudantes estavam vivenciando problemas psicológicos relacionados à depressão e ansiedade, além de disfunção social. Os resultados também revelaram falta de conscientização sobre aplicativos de saúde mental e incerteza quanto ao uso de tais aplicativos.	Melhorar a alfabetização em saúde digital dos estudantes, aumentando sua conscientização sobre aplicativos de saúde mental e os métodos de tratamento e benefícios destes.

As amostras dos estudos empíricos variaram entre o mínimo de 52 estudantes de uma universidade em Bangladesh^(6)^ e o máximo de 746.217, em estudo sobre estudantes chineses^(7)^. No total, as amostras são de 1.089.922 estudantes universitários em contexto global. Os estudantes eram de diversos cursos na graduação, porém foram mais frequentes pesquisas voltadas aos estudantes da área da saúde.

Foram obtidos estudos de 16 países. Os Estados Unidos da América apresentam mais estudos sobre a saúde mental dos universitários, com um total de cinco (19,25%), seguido da China, com quatro (15,4%) estudos. O Brasil, Bangladesh e a Turquia trazem dois (7,7%) estudos cada. Os demais países, como Alemanha, Arábia Saudita, Emirados Árabes Unidos, Espanha, França, Indonésia, Iraque, Itália, Japão, Peru e Ucrânia, apresentam um (3,85%) estudo cada. Em relação à metodologia de cada estudo, são nove (34,65%) qualitativos, seis (23,1%) quantitativos, oito (30,8%) descritivos e três (11,55%) estudos prospectivos/observacionais.

Por meio da análise integral dos 26 artigos selecionados, foi possível identificar que os principais focos foram organizados em temas e subtemas, conforme sintetizados narrativamente a seguir.

### Estado de saúde mental dos universitários

Este subtema é composto por 14 artigos^(2, 4, 8, 9, 10, 11, 12, 13, 14, 15, 16, 17, 18, 19)^. Os estudos analisam o estado da saúde mental dos estudantes universitários no período pandémico, e apresentam propostas de intervenção para questões psicológicas consequentes da doença. No total, 336.579 estudantes foram observados.

Do total de estudos, cinco encontraram, entre os estudantes observados, a prevalência de níveis moderados a graves de ansiedade e depressão em consequência da pandemia de COVID-19. Os maiores níveis de ansiedade e depressão estão associados a uma diminuição da saúde mental e a maiores índices de pensamentos suicidas na pandemia. Ainda, os níveis de ansiedade e depressão subiram no período pandémico, e o bem-estar mental sofreu reduções, justamente devido ao isolamento e à incapacidade dos estudantes de lidar com situações de estresse^(8, 9)^. Os níveis de estresse também se mostraram significativamente elevados, contribuindo para uma baixa satisfação com a vida^(10)^. Certos autores^(9)^ perceberam que estudantes que passam mais de três horas por dia navegando por informações sobre a COVID-19 também estão predispostos a aumentar os níveis de ansiedade. Por outro lado, outros autores^(11)^ identificaram que o estresse mental de alguns universitários durante a pandemia de COVID-19 reduziu, embora tenham apresentado aumento da carga psicológica. A ansiedade e o estresse estão mais presentes no sexo feminino, e indivíduos com alto altruísmo conseguem equilibrar os níveis negativos dessas condições^(10, 12)^.

Os autores supracitados defendem uma intervenção com foco na redução dos níveis de ansiedade e depressão através de atividades que permitam a interação e apoio psicológico remoto aos estudantes, que lhes possibilitem manter a realização de atividades físicas e que lhes promovam educação de qualidade, satisfação das necessidades básicas e ações de autocuidado.

Ainda, em seis artigos, concluiu-se que a pandemia de COVID-19 trouxe impactos relacionados a outras condições de saúde e qualidade de vida, o que contribuiu para a diminuição da saúde mental dos universitários. A preocupação com a saúde e com o desempenho acadêmico, o medo de infecção e oferta inadequada de alimentos, a dificuldade de concentração, as interrupções nos padrões de sono, as diminuições nas interações sociais e a ausência de atividades físicas e atividades recreativas são identificados como efeitos negativos ao psicológico consequentes da pandemia^(2, 13)^. Com a pandemia, os níveis de ansiedade, depressão, estresse, angústia, tristeza, preocupação e pânico foram acentuados, impactando diretamente a saúde mental dos estudantes^(4, 14)^. Ainda, a pandemia afetou outras condições de saúde e qualidade de vida, como a qualidade do sono, bem-estar e vida sexual, que se tornaram inferiores, assim como os estudantes se tornaram mais desanimados, propensos a pensamentos suicidas e autodestrutivos, com chances de desenvolverem estresse pós-traumático^(4, 15)^.

Foi identificado que estudantes que percebem a pandemia como possibilidade de crescer na carreira e trabalhar por conta própria apresentam níveis médios a altos de otimismo, proatividade, satisfação da necessidade psicológica e intenção empreendedora, o que contribui para a redução de problemas de saúde mental^(16)^. Em geral, os autores supracitados defendem intervenções e estratégias preventivas abordando a saúde mental, com foco em melhorar a qualidade de vida e promover bem-estar emocional através de apoio psicológico, atividades físicas e sono adequado, além de apoio universitário, para garantir que os objetivos acadêmicos sejam alcançados.

Neste sentido, dois artigos trataram da saúde mental dos estudantes universitários antes e durante a pandemia de COVID-19, identificando que houve aumento significativo dos estressores associados e diminuição da saúde mental. Em um artigo^(17)^, foram identificados níveis significativamente mais elevados de depressão, ansiedade e estresse nos universitários durante o período pandêmico em comparação ao período anterior à pandemia. No outro artigo^(18)^, não foram encontrados níveis significativamente alterados de transtornos mentais nos estudantes, ao serem observados nos anos de 2018, 2019 e 2020. Estudantes que moravam sozinhos ou com colegas de quarto tiveram essas condições mais acentuadas do que estudantes que moravam com os pais e que tinham acesso a um espaço interno com quintal e jardim. Embora certos autores^(18)^ não tenham identificado alterações significativas, outros^(17)^ sugerem que cada estudante seja acompanhado individualmente para a identificação da necessidade psicoterapêutica que ele possa apresentar e que a universidade promova um atendimento psicológico segundo cada necessidade.

Acrescenta-se que um artigo trouxe dados apontando que a COVID-19 contribuiu para o sofrimento psicológico de diversos estudantes. Os estudantes universitários LGBT sofreram grandes impactos psicológicos no período pandêmico pelo fato de que muitos já enfrentam ansiedade e depressão pela não aceitação e apoio de sua identidade por seus familiares, condições que se agravaram com a pandemia^(19)^. Os autores defendem intervenções com apoio psicológico durante a pandemia para a saúde mental^(19)^.

### Estressores e fatores associados aos problemas de saúde mental

Inclusos nesta categoria estão quatro estudos^(6, 7, 20, 21)^, com uma amostragem total de 748.878 estudantes universitários. Desses, três artigos apresentaram os fatores mais comuns encontrados entre estudantes universitários que contribuem para o aumento do risco de problemas de saúde mental no período pandêmico. Estudantes com mais tempo de curso, localizados em áreas rurais ou na periferia, com histórico de bebida alcoólica, baixo suporte social, com parentes ou conhecidos infectados e estudantes do sexo feminino estão mais sujeitos a desenvolverem problemas de saúde mental em consequência da pandemia^(6, 7)^. A ansiedade e a depressão em nível leve estão entre as primeiras condições a aparecer nesses estudantes, bem como o estresse agudo^(7)^. Os problemas de saúde mental mais comuns entre os universitários são, primeiramente, a ansiedade, seguida de depressão e estresse. Questões relacionadas à vida acadêmica – incertezas sobre o programa acadêmico e realização pessoal, mudanças no formato de ensino –, relacionadas à saúde – saúde pessoal, de amigos e familiares –, relacionadas à disponibilização de informações confiáveis sobre a COVID-19 e relacionadas à falta de apoio social também são fatores considerados^(20)^. Os autores defendem como intervenções apoio psicológico com foco no bem-estar mental.

Outro estudo^(21)^ identificou que o sofrimento psicológico é uma consequência que pode surgir em uma grande porcentagem de estudantes que desenvolvam problemas de saúde mental. Logo, sugerem que as universidades devem apoiar iniciativas de saúde mental e educacionais direcionadas ao aumento da autoestima e autoeficácia.

### Impacto da espiritualidade e do sentido da vida na saúde mental dos estudantes

Destacam-se quatro estudos^(22, 23, 24, 25)^ inclusos nesta categoria, que investigam a relação entre o significado da vida durante a pandemia de COVID-19 e a saúde mental em 2.6,2 estudantes universitários. Desse total, dois artigos identificaram efeitos positivos de manter um sentido da vida para a melhora da saúde e bem-estar mental. Estudantes que traçaram trajetórias significativas do sentido da vida em período pandêmico, mantendo sua espiritualidade, melhor puderam enfrentar o sofrimento psicológico e manter sua saúde mental^(22, 23)^. Ainda, a espiritualidade esteve associada à empatia^(23)^. Os autores sugerem, então, como intervenções para problemas de saúde mental, abordagens e aconselhamentos centrados no significado da vida e estratégias espirituais.

De acordo com dois artigos, há uma estreita relação entre o sentido da vida e a saúde mental. A presença de sentido da vida está associada a níveis mais baixos de depressão, ansiedade e estresse^(24)^. Como um complemento a essa observação, a preocupação com a COVID-19 traz efeitos negativos no significado na vida e resiliência dos universitários, além de contribuir para o aparecimento de transtornos de saúde mental^(25)^. Logo, os autores sugerem programas de intervenção com foco no sentido da vida, atenção plena e resiliência, que são pontos fortes para reduzir os estressores e melhorar a saúde mental.

### Relação entre atividade física e saúde mental

Esta temática foi discutida por três estudos^(26, 27, 28)^, compreendendo 1.983 estudantes universitários, que foram investigados sobre a prática de atividades física durante a pandemia e seu impacto na saúde mental e bem-estar desses indivíduos.

Em dois artigos, houve a conclusão de que a prática de exercícios regulares contribui para a redução dos níveis de ansiedade, depressão e estresse e, consequentemente, melhora da saúde mental, já que o corpo libera hormônios que levam o organismo a uma sensação de bem-estar. Alguns autores^(26)^ analisaram um grupo fisicamente ativo e um inativo de estudantes, e observaram que o grupo inativo apresentou maiores escores de ansiedade e depressão, e identificaram que o estresse aumentou em estudantes que passaram as primeiras semanas de isolamento sem praticar atividades físicas. Outros^(27)^ perceberam que os exercícios contribuem para positivos estados de humor nos estudantes universitários, além de estarem associados ao consumo de frutas, verduras e peixes. Por outro lado, a depressão e baixos níveis de saúde mental estiveram associados ao consumo de cereais, leguminosas e carnes com baixo teor de gordura, além do sedentarismo. Os autores defendem a prática de atividades físicas e alimentação saudável para a melhora dos níveis de saúde mental dos estudantes.

Ainda foi identificado que a ansiedade provocada pela COVID-19 esteve fortemente relacionada ao exercício compulsivo, bem como à DE, e isso foi mais comum em estudantes com menor intolerância à incerteza^(28)^. Dessa forma, os autores sugerem esforços de prevenção e intervenção direcionados à intolerância, à incerteza e à ansiedade, além de intervenções destinadas a gerenciar o sofrimento psicológico^(26, 27, 28)^.

### Saúde mental e ensino digital

Em um estudo^(29)^ incluso nessa categoria, 154 estudantes universitários foram observados para a compreensão de como a substituição do ensino presencial para o ensino remoto por conta da pandemia impactou a sua saúde mental. A pesquisa^(29)^ investigou a atitude de estudantes sobre o uso de aplicativos digitais, e identificou que muitos se sentiam inseguros e incertos quanto a esse uso pela falta de conscientização e conhecimento sobre aplicativos. Dessa forma, os autores sugeriram melhorar a alfabetização dos estudantes em saúde digital, aumentando sua conscientização e lhes apresentando os benefícios dos aplicativos.

## DISCUSSÃO

Por meio desses estudos, descobriu-se a prevalência da ansiedade, depressão e estresse entre os estudantes por conta da pandemia, contribuindo para o aumento dos riscos de desenvolverem problemas de saúde mental. A princípio, constatou-se que o desenvolvimento de problemas e transtornos psicológicos consequentes da pandemia é específico a cada estudante e suas diferentes condições. Alguns autores^(18)^ concluíram que a pandemia trouxe efeitos mínimos à saúde mental dos estudantes. Alguns^(11)^ perceberam que, ao invés de um aumento ou inalteração dos problemas de saúde mental, houve uma redução no estresse mental de alguns universitários durante a pandemia. Outros^(24, 25)^ também observaram efeitos mínimos na saúde mental de estudantes que mantiveram um significado para a vida e sua espiritualidade em período pandêmico, sendo observados, no máximo, níveis leves de depressão em poucos estudantes.

Por outro lado, demais autores^(10, 17, 20)^ perceberam que a pandemia elevou de forma significativa os níveis de ansiedade, depressão e estresse desses indivíduos, levando-os ao desenvolvimento de distúrbios de saúde mental, aparecimento de transtornos mentais e diminuição do bem-estar. Outros^(4, 14)^ apontam que, além da ansiedade, depressão e estresse, condições de saúde mental mais frequentemente encontradas nos estudantes em consequência da pandemia, a angústia, a tristeza, a preocupação com a vida e o desenvolvimento acadêmico, o medo e o pânico tiveram seus níveis acentuados, igualmente contribuindo para a redução da saúde mental.

Grande parte dos estudos observou que os problemas de saúde mental são mais comuns ao sexo feminino. Eles concluíram que as condições de ansiedade, depressão e estresse são mais comumente encontradas entre as mulheres estudantes universitárias^(6, 7, 10, 12, 16, 17)^.

Um dos fatores de risco observados, relacionados ao aumento dos problemas de saúde mental, é passar algumas horas do dia navegando por informações e obtendo maiores conhecimentos sobre a COVID-19, o que leva a altos níveis de ansiedade^(9)^. Certos estudos^(2, 6, 7, 13, 26, 29)^ também apontam como fatores de risco a preocupação com a saúde própria e a saúde da família e amigos, o medo de infecção, o medo da falta de alimentos, a preocupação com o desempenho acadêmico, a dificuldade de concentração, as interrupções nos padrões de sono, as diminuições nas interações sociais, a ausência de atividades físicas e atividades recreativas, morar sozinho ou com colegas de quarto, ter mais tempo de curso, morar em áreas rurais ou na periferia, ter histórico de bebida alcoólica, ter baixo suporte social, mudanças no formato de ensino, ter dificuldades financeiras e técnicas no uso de plataformas digitais, sofrer isolamento em redes sociais, insegurança e incerteza no uso de aplicativos de ensino no período pandêmico, inatividade, e sedentarismo.

Os altos níveis de ansiedade, depressão e estresse estão associados a maiores índices de pensamentos suicidas e auto-destrutivos na pandemia, além de uma má qualidade de sono, de bem-estar e de vida sexual, provocando desânimo e chances de que os estudantes desenvolvam estresse pós-traumático^(4, 8, 15)^. Os problemas de saúde mental também podem levar ao sofrimento psicológico e demais problemas alarmantes do estado psicológico, além de levar à necessidade de praticar exercícios físicos de forma compulsiva e a uma DE^(19, 21, 28)^.

Diante das consequências provocadas pela pandemia de COVID-19 na saúde mental dos estudantes universitários, alguns estudos se dedicaram a apresentar estratégias de enfrentamento e superação aos fatores de risco associados ao aumento das condições de ansiedade, depressão e estresse. A prática de atividades físicas foi identificada como uma das melhores práticas para manter a saúde mental em tempos de COVID-1 9^(26, 27)^. Os autores observaram que os exercícios contribuem para níveis mais baixos de escores de ansiedade, depressão e estresse, ajudam o bem-estar mental, melhoram o humor e estão associados à uma alimentação mais saudável, que também é fator importante para a saúde mental^(26, 27)^. Outros autores^(22, 23)^ defendem que a espiritualidade também é um fator que contribui para o equilíbrio da saúde mental, pois possibilita que os estudantes encontrem um significado e sentido para a vida, mesmo em meio ao caos pandêmico. Ainda defendem a importância da aceitação, auto distração e *coping* (enfrentamento) ativo como estratégias de enfrentamento. Em um contexto geral, propostas de intervenções contra o comprometimento psicológico foram observadas nos artigos discutidos, destacando, principalmente, estratégias de apoio psicológico e emocional aos estudantes.

Estudos que investigaram uma maior quantidade de estudantes universitários, acima de 500 participantes, por exemplo, trouxeram dados mais específicos sobre as consequências da pandemia de COVID-19 à sua saúde mental, demonstrando que, de fato, houve prejuízo a uma parte considerável dos estudantes do mundo, que apresentaram altos níveis de ansiedade, depressão e estresse. Esses mesmos dados não foram observados em vários dos estudos que investigaram uma quantidade menor de estudantes, abaixo de 100, por exemplo. Nestes, os dados apontaram poucas alterações psicológicas e mínimas consequências à saúde mental. Deve-se considerar, porém, a dificuldade de se obter dados mais precisos com poucos participantes em uma pesquisa, já que muitas vezes as condições socioeconómicas entre eles podem não se diferir de forma significativa. O mesmo não ocorre com pesquisas que envolvem milhares de participantes, pois as chances de que possuam amplas diferenças socioeconómicas, de vivência e de saúde são maiores, e isso impacta os resultados da pesquisa. Ainda, é importante considerar as diferenças de países, por se tratar de estudo de contexto global. Pesquisas com estudantes em países mais pobres e com menos condições de enfrentamento à pandemia tendem a apresentar resultados diferentes de pesquisas realizadas com estudantes em países e universidades com melhor preparo para a situação atual.

Com esta revisão de escopo, constatou-se que existem evidências consideráveis sobre a questão na literatura nacional e internacional, porém o maior foco desses estudos foram os estudantes universitários chineses, investigados em maior quantidade. Isto pode sugerir que uma futura revisão sistemática possa ser apropriada para a investigação da saúde mental dos estudantes universitários na China. No entanto, há carência de evidências mais completas relacionadas aos demais países, o que implica a exigência de dados mais específicos, e não aponta a necessidade de revisões sistemáticas para o conteúdo.

### Limitações do estudo

A presente revisão de escopo é restrita apenas à busca por evidências, por isso a seleção dos estudos incluídos foi submetida a viés de critérios de inclusão e exclusão específicos, bem como à interpretação subjetiva dos pesquisadores.

### Contribuições para as áreas da saúde e políticas públicas

Esta revisão oferece uma compreensão mais aprofundada dos fatores que afetaram a saúde mental dos estudantes universitários durante o período da pandemia de COVID-19, podendo, então, oferecer *insights* para o desenvolvimento de estratégias de intervenção e apoio direcionados. Ainda, foram oferecidas perspectivas sobre intervenções eficazes que podem ser implementadas para mitigar os impactos negativos da pandemia.

O estudo também pode contribuir para a atuação dos profissionais de saúde mental, educadores e formuladores de políticas, pois, ao destacar os desafios enfrentados pelos estudantes universitários e as possíveis intervenções, orienta a tomada de decisão e o desenvolvimento de programa de suporte adequado às necessidades específicas dessa população.

## CONSIDERAÇÕES FINAIS

A revisão de escopo apresentada sublinha a relevância de estudar a saúde mental dos estudantes universitários, destacando a necessidade urgente de desenvolver estratégias de cuidado e proteção neste âmbito. A pandemia de COVID-19 acentuou significativamente os estressores enfrentados por esses estudantes, exacerbando questões de bem-estar mental. Esse aumento nos fatores estressores é refletido em sintomas como ansiedade, depressão e sensação de isolamento, que se tornaram mais prevalentes durante a pandemia.

Como estratégias de enfrentamento, sugere-se que a incorporação de atividades físicas regulares, uma alimentação saudável, a prática da espiritualidade e a busca por um sentido na vida podem servir como âncoras importantes para muitos estudantes, oferecendo um caminho para encontrar paz e propósito, mesmo em tempos difíceis. O apoio psicológico e emocional, seja através de aconselhamento profissional ou de redes de suporte social, também é crucial para ajudar os estudantes a navegar por essas experiências desafiadoras.

Embora a literatura existente já forneça dados relevantes para a discussão, ainda há uma falta de informações específicas em relação a alguns países, o que impede uma compreensão completa do problema em uma escala global. Essa lacuna sugere a necessidade de pesquisas mais abrangentes e detalhadas que possam capturar as nuances das experiências dos estudantes em diferentes contextos culturais e sociais.

A saúde mental dos universitários foi severamente impactada pela pandemia, e a criação de políticas e programas de apoio psicológico é crucial. Instituições de ensino devem se engajar ativamente na implementação de serviços de saúde mental, oferecendo suporte adequado e acessível para seus alunos. Além disso, há uma necessidade de conscientização contínua sobre a importância da saúde mental, não apenas em tempos de crise, mas como uma parte integral do bem-estar dos estudantes em geral. A priorização da saúde mental é essencial para garantir que os estudantes possam enfrentar os desafios acadêmicos e pessoais com resiliência e apoio adequado.
